# A Better Understanding of the Clinical and Pathological Changes in Viral Retinitis: Steps to Improve Visual Outcomes

**DOI:** 10.3390/microorganisms12122513

**Published:** 2024-12-05

**Authors:** Nghi M. Nguyen, Christopher D. Conrady

**Affiliations:** 1 Department of Ophthalmology and Visual Sciences, University of Nebraska Medical Center, Omaha, NE 68198, USA; 2 Department of Pathology, Microbiology, and Immunology, University of Nebraska Medical Center, Omaha, NE 68198, USA

**Keywords:** viral retinitis, retinal inflammation, herpes simplex virus, cytomegalovirus, acute retinal necrosis

## Abstract

Infectious retinitis, though rare, poses a significant threat to vision, often leading to severe and irreversible damage. Various pathogens, including viruses, bacteria, tick-borne agents, parasites, and fungi, can cause this condition. Among these, necrotizing herpetic retinitis represents a critical spectrum of retinal infections primarily caused by herpes viruses such as varicella-zoster virus (VZV), herpes simplex virus (HSV), and cytomegalovirus (CMV). This review underscores the retina’s susceptibility to viral infections, focusing on the molecular mechanisms through which herpetic viruses invade and damage retinal tissue, supported by clinical and preclinical evidence. We also identify existing knowledge gaps and propose future research directions to deepen our understanding and improve therapeutic outcomes.

## 1. Introduction

Infectious chorioretinitis is an inflammatory condition of the retina caused by viral, bacterial, fungal, or parasitic infections that disrupt retinal function and lead to significant vision loss [1,2]. Among the viral pathogens, herpes simplex virus (HSV), varicella-zoster virus (VZV), and cytomegalovirus (CMV) are the most common pathogens [3,4,5]. These viruses are associated with a spectrum of ocular diseases, including herpetic keratitis, chronic anterior uveitis, acute retinal necrosis, and progressive outer retinal necrosis [3,4,6]. Both ARN and PORN can lead to serious complications, such as optic neuropathy, chronic retinal ischemia, and retinal detachment [3,4]. While ARN generally occurs in immunocompetent individuals, PORN is more frequently seen in immunocompromised patients and was first recognized during the HIV/AIDS epidemic [3,4].

Viral retinitis is considered rare, with HSV retinitis affecting fewer than 1000 people annually in certain regions, and if these rates are extrapolated to the US population, there are likely 250 new cases a year within the US [7,8]. However, the global incidence may be higher, particularly in areas with an elevated HSV-1 prevalence and limited access to diagnostic and treatment resources [9]. Another retinopathy associated with herpesvirus infection is CMV retinitis, which often occurs in immunocompromised populations such as those with HIV/AIDS, bone marrow or solid organ transplants, those undergoing chemotherapy, or those taking immunosuppressants for autoimmune conditions [10,11]. Before the advent of highly active antiretroviral therapy (HAART), CMV retinitis affected nearly one-third of AIDS patients [3,4,12,13].

This review aims to provide a comprehensive analysis of viral retinitis, with a particular focus on comparing two prominent causative agents, alpha herpesviruses (HSV and VZV) and CMV. We will begin by outlining the structure of the retina, its homeostatic mechanisms, and how the retinal immune response is regulated under normal conditions. Next, we will examine the clinical presentations and management of retinopathy associated with viral infections while also addressing the current in vivo models used to study viral retinitis. Following this, we will dive deeper in discussing herpetic viruses in retinitis, focusing on the pathogeneses of HSV, VZV, and CMV, as well as their strategies for evading the innate immune system. Throughout the review, we will highlight the limitations in our current understanding and suggest potential directions for future research to bridge existing knowledge gaps.

## 2. Overview of the Retina, Retinal Homeostasis, and Retinal Immune Response Regulation

### 2.1. Overview of the Retina, Retinal Barriers, and Immune Privilege

The eye is a sophisticated sensory organ that responds to light and enables vision. Light first passes through the cornea at the front of the eye, where it is focused by the lens towards the retina, which resides at the back of the eye. The retina, a complex tissue composed of multiple layers of cells, plays a key role in visual processing. It contains ten distinct layers of neurons interconnected by synapses, primarily classified into the following three types: photoreceptor, neuronal, and glial cells. Visual information is transmitted to the brain via the optic nerve [14,15]. At the core of the retina’s architecture are several layers, including the inner limiting membrane, the nerve fiber layer, and the ganglion cell layer, extending outwards to the retinal pigment epithelium (RPE). The RPE, a layer of polarized epithelial cells positioned between the retina and the choroid, is critical for the survival of the neurosensory retina. It regulates nutrient transport and homeostasis [16,17,18]. Additionally, the RPE supports photoreceptor health by phagocytosing and degrading spent photoreceptor segments and shielding the retina from light-induced and oxidative damage [19]. Due to its high metabolic demands, the RPE is particularly susceptible to oxidative stress, a major risk factor for retinal degeneration, necessitating rapid mitigation to preserve vision [18,19,20,21]. The RPE employs autophagy and mitophagy to manage oxidative stress by directing reactive oxygen species (ROS)-producing mitochondria to lysosomes, thereby reducing ROS production [18,19,20,21]. Furthermore, the tight junctions between RPE cells form the outer blood–retinal barrier (BRB), which isolates the retina from the choroidal circulation. This barrier limits the penetration of pathogens and large molecules by expressing surface ligands and soluble factors that inhibit immune activation, thereby contributing to the eye’s immune privilege [22,23,24]. Moreover, inhibitory signals from the RPE can trigger the death of active immune cells or transform them into immunosuppressive or regulatory cells [22,25].

To protect the retina, several protective strategies have evolved, including immune privilege [14]. Specific compartments within the eye, such as the anterior chamber, vitreous cavity, and subretinal space, are immune-privileged areas where exposure to foreign antigens may ultimately lead to tolerance rather than immune activation [24,26,27,28]. The physical barriers, such as the BRBs, consist of the following three components: (1) inner blood–retina barrier (iBRB), (2) outer blood–retinal barrier (oBRB), and (3) blood–aqueous barrier, which, together, help to insulate the retina from the systemic circulation, minimizing the risk of pathogen invasion and systemic inflammation [24,26,27,28]. The neurovascular unit, composed of vascular endothelial cells, pericytes, glial cells, and neurons, maintains the integrity of the iBRB [29,30].

The retina also utilizes neuroimmune regulatory proteins to suppress inflammation and tolerate pathogens’ negative impacts on host tissue fitness without directly affecting the pathogen burden. Specifically, these regulatory proteins play critical roles in modulating microglia, macrophage, T-cell, and complement activation and inducing immune cell death via various specialized pathways, as reviewed by Murakami and colleagues [31]. These mechanisms contribute to maintaining the retina’s immune privilege. Research into these immune regulatory processes, particularly those involving effector cells like immune and RPE cells, has largely focused on this second line of defense. However, whether the retina can actively control pathogens—for example, restricting or neutralizing their toxic effects—remains uncertain.

### 2.2. The Innate Immunity

Inflammation is an elicited response to harmful stimuli such as pathogen entry, damaged cells, toxins, or irradiation. It is crucial in alerting the body to injury and initiating the healing process, making it an essential defense mechanism. However, prolonged and uncontrolled inflammation can damage tissues, and the retina, in particular, has a limited capacity for renewal and repair [26,27]. This makes it highly vulnerable to both internal and external insults. When pathogens bypass the BRBs and invade the retina, local immune responses activate to minimize damage and restore balance. Resident retinal immune cells, such as microglia, as well as the complement system, help to resist transient threats (para-inflammation) [32]. Retinal microglia reside strategically between the inner plexiform layer (IPL) and the outer plexiform layer (OPL) to help maintain proper synaptic connectivity in these two locations. The entrance of pathogens or the activation of damage- or pattern-associated molecular patterns (DAMPs/PAMPs), recognized by pathogen recognition receptors (PRRs), activates microglia and other retinal cells, including the RPE [33]. In other neurological tissues, the Triggering Receptor Expressed on Myeloid cells 2 (TREM2) and DNAX activation protein 12 (DAP12) initiate the signal transduction cascades to enhance microglia activation and phagocytosis [34,35]. Additionally, signaling molecules produced by retinal neurons—cytokines (e.g., TGF-B), chemokines (e.g., CX3CL1), and neurotrophic growth factors—regulate microglial activation [36,37,38,39]. When the threat surpasses a critical threshold, infiltrating immune cells can further support the retina by either inducing cell death or transforming them into immunosuppressive cells to mitigate immunopathology [40].

**Figure 1:**
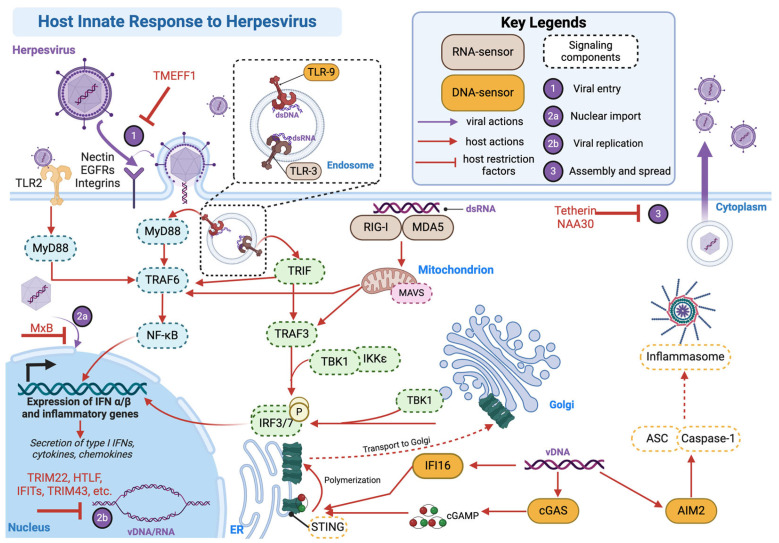
Host innate response to herpesviruses. Herpesviruses are DNA viruses that are detected by multiple host DNA and RNA innate immune sensors at different stages of their infection and replication cycle to activate proinflammatory cytokine production and antiviral type I interferons. Arrows connect pathways; blunted lines represent host restriction factors that inhibit various stages of the viral life cycle. This figure was created using Biorender.com (accessed on 1 December 2024). IFN, interferon; vDNA, viral DNA.

Viral infections activate innate immune responses and cell death pathways that limit viral spread and contain inflammation. Healthy retinal cells maintain a non-inflammatory state, but infection or stress-induced cell death can amplify immune responses. Infected cells undergoing lytic cell death release cytokines and intracellular contents, which activate immune cells through PRRs like toll-like receptors (TLRs) and nod-like receptors (NLRs), subsequently leading to the production of type I interferons (IFN), interferon-stimulated genes (ISGs), cytokines, and chemokines (Figure 1). Furthermore, intracellular innate immunity involves co-evolved antiviral restriction factors (RFs) that specifically inhibit infecting viruses at multiple states of viral cycles to provide an additional early defense. For instance, the TMEFF1 protein can interact with Nectin-1 to prevent herpes simplex virus from entering neuronal cells [41,42]. The host also produces RFs at the viral gene production stage, including nuclear import, transcription, replication, and translation (reviewed in Ref. [43]). Other RFs act during the viral gene production, replication, and assembly stages; for example, tetherin and SERINC5 reduce virus release by disrupting particle assembly and egress [44,45].

The host cell also possesses DNA and RNA sensors to detect viral genetic material. Specifically, RIG-I-like receptors (RLRs), melanoma differentiation-associated protein 5 (MDA-5), and TLR3 can recognize viral double-stranded RNA (dsRNA) (Figure 1). Meanwhile, cyclic GMP-AMP synthase (cGAS) and interferon-gamma inducible protein 16 (IFI16) are common viral DNA sensors that transmit signaling through STING (stimulator of interferon genes), while AIM2 is the DNA sensor that recruits factors to activate the inflammasome, an essential component of antiviral defense (reviewed in Refs. [46,47]) (Figure 1). Currently, knowledge of whether the retina utilizes similar PRRs, viral sensors, and RFs for viral defense and homeostasis is limited. Studies have shown that retinal cells, including Müller cells, RPE cells, and microglia, express various PRRs. For instance, RPE cells express TLR3 and TLR9, while Müller cells express TLR7 and TLR9 [48,49]. Figure 1 summarizes the host response to herpesvirus entry into the cell.

## 3. Viral Retinitis and Current Animal Models

### 3.1. Clinical Presentations and Management of Viral Retinitis

**Figure 2:**
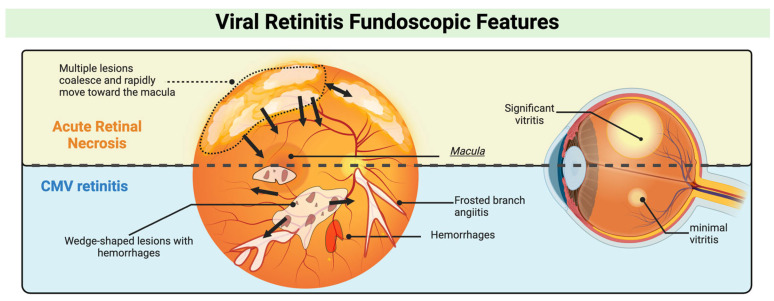
Viral retinitis ophthalmic features. The clinical presentations of ARN and CMV retinitis are typically different, with classic cases of ARN presenting in the peripheral retina as yellowish chorioretinal lesions that spread posteriorly towards the macula (left upper side) with substantial intraocular inflammation (right upper side). On the other hand, classic CMV retinitis is seen as wedge-shaped areas of retinal whitening and hemorrhages centered around blood vessels that expand out from the vessels with minimal to no overlying vitritis (lower left and right side, respectively). This figure was created using Biorender.com (accessed on 1 November 2024).

Acute retinal necrosis (ARN), progressive outer retinal necrosis (PORN), and CMV retinitis are infectious-disease-associated forms of retinopathy from viral infections. The viral agents that are causative for these diseases belong to the *Herpesviridae* family, including HSV-1 and HSV-2, VZV, and CMV. ARN is characterized by the following: (1) scalloped areas of patchy necrosis in the peripheral retina, (2) the rapid circumferential expansion and coalescence of necrotic regions, (3) occlusive vasculopathy, and (4) significant inflammation in both the vitreous and aqueous humor (Figure 2) [3,4]. Most ARN cases affect only one eye, though bilateral ARN (BARN) occurs in up to a third of patients. Symptoms may be mild and nonspecific, such as eye redness, light sensitivity (photophobia), floaters, blurred vision, and pain in the early stages of the disease.

The clinical presentation of ARN can be divided into two distinct phases. Patients often exhibit an anterior chamber reaction in the early phase, commonly accompanied by fine or mutton fat keratic precipitates. Dilated fundoscopy may reveal varying levels of vitreous inflammation and characteristic multifocal patches of yellowish-white infiltrates located within the retina (Figure 2) [3,50]. These features typically originate in the peripheral retina, often with signs of vasculitis (Figure 2). As the disease progresses, the necrotic damage worsens, accompanied by more pronounced vitreous inflammation. The peripheral retinal lesions rapidly extend toward the posterior pole and coalesce, leading to retinal atrophy and vitreous traction, contributing to high rates of retinal detachment (RD) and poor visual prognoses [3,51,52]. Although antiviral agents, including acyclovir, valacyclovir, famciclovir, foscarnet, and ganciclovir, given systemically and/or locally, are utilized to slow ARN progression, reduce RD incidence, and minimize the risk of CNS and other eye involvement, up to 60% of patients still develop RD. While no randomized control trials exist, we advocate for both local and initial high-dose systemic antiviral therapy due to its good intraocular penetrance and the initiation of both topical and systemic steroids to inhibit viral infection as rapidly as possible, minimize additional retinal immunopathology, and reduce the risk of CNS involvement. Although likely underreported, ARN may coincide with herpetic meningoencephalitis, manifesting as seizures, fever, or confusion, prompting further investigation for CNS involvement [53]. There is a broad upregulation of proinflammatory and vascular mediators, including IL-6, IL-8, IL-18, MCP-1, MIF, eotaxin, IP-10, and soluble ICAM-1, in the aqueous humor and serum samples from ARN patients, compared to controls. Additionally, IL-10, an anti-inflammatory cytokine, is significantly elevated in ARN cases relative to non-inflammatory controls [54]. Diagnosing ARN can be challenging due to its asymptomatic nature in the early stages [50]. Additionally, we advocate for the liberal use of molecular diagnostics with the aqueous or vitreous fluid, and cerebrospinal fluid in cases of suspected CNS involvement, such as polymerase chain reaction to confirm the specific offending pathogen in patients suspected of having ARN due to other, less common pathogen mimickers [1]. Unfortunately, while systemic inflammatory changes are evident in patients with ARN, systemic serologic testing is often unrevealing due to the high rates of seropositivity for HSV and VZV within the general population. In fact, a negative IgM and IgG may be more revealing, as they would be more supportive of an alternative diagnosis.

PORN is also a rapidly progressive necrotizing herpetic retinitis, typically seen in highly immunocompromised patients, primarily caused by VZV infection [3,4]. While its clinical features resemble those of ARN, PORN is distinguished by the following five key characteristics: (1) involvement of the outer retina; (2) a lack of significant inflammation in the vitreous and aqueous humor; (3) less retinal vasculature involvement; (4) higher rates of concurrent CNS involvement; and (5) most have a history of prior cutaneous zoster. Notably, PORN may progress despite systemic antiviral therapy, frequently leading to blindness within days of onset, as it more commonly starts within the macula [3,4]. Similar to PORN, CMV retinitis occurs almost exclusively in locally or systemically immunocompromised patients [55], though, in very rare cases, CMV retinitis can also be found in immunocompetent patients [56]. Both PORN and CMV retinitis are commonly seen in HIV-positive patients with CD4+ T-cell counts below 50–100 cells/µL [3,4,13]. Due to the significant advances in antiretroviral therapy (ART), the incidence of CMV retinitis and PORN has substantially declined in high-income countries. However, while these complications are now rare in HIV-positive patients, they can still occur in individuals who have undergone hematopoietic stem cell transplants or solid organ transplants, where immunosuppressive therapy increases the risk of CMV reactivation and retinitis [57,58,59]. While CMV retinitis typically progresses slower than PORN and ARN, it can still lead to retinal detachment and blindness if left untreated. Compared to ARN, CMV retinitis presents with reduced or absent intraocular inflammation linked to the patient’s compromised immune status (Figure 2). This retinitis advances more gradually, and its subtle symptoms may lead to a delayed diagnosis. Patients with CMV retinitis typically present with painless vision loss and eye findings such as slowly progressive perivascular white retinal infiltrates, retinal vasculitis with a frosted branch angiitis pattern, and/or multiple retinal hemorrhages (Figure 2). Distinct clinical forms have been described as fulminant, granular, or perivascular CMV retinitis, with the perivascular presentation being the most “classic” and common. A notable clinical feature is the absence of significant vitreous inflammation, leaving a clear view of the retina. This contrasts with other posterior chamber infectious disorders such as ocular toxoplasmosis, where vitritis is common, hemorrhages are typically rare, and retinochoroidal lesions are typically whiter than those seen in CMV. In a clinical study on HIV-positive patients in India, HIV patients who developed CMV retinitis while on ART (ART-failure patients) had less severe disease, but more often experienced bilateral eye involvement compared to ART-naïve patients [60]. A visual illustration of the clinical findings for ARN and CMV retinitis is described in Figure 2.

Currently, no therapeutic strategy eradicates the viral infection (virus returns to latent state) caused by herpesviruses and restores the retinal damage caused by these viruses. The current available management for herpesvirus infection is the use of antiviral agents (e.g., acyclovir, valacyclovir, famciclovir, foscarnet, or ganciclovir), recommended by the American Academy of Ophthalmology to minimize retinal necrosis and progression to RD [61]. While these antiviral therapies can mitigate some damaging sequelae of herpesvirus infections, they are often insufficient regarding the development of substantial retinal pathology, even with aggressive therapy, likely owing to a pronounced immune response to the infection. Notably, the progression rate of viral retinitis varies significantly depending on the type. ARN and PORN typically progress much more rapidly than CMV retinitis, with the potential that ARN and PORN may lead to total retinal necrosis within days if untreated. Moreover, some reports have noted that these treatments can lead to developing drug resistance in immunocompromised patients, especially CMV if used long-term [62,63,64].

### 3.2. Current In Vivo Models for Viral Retinitis

To improve the treatment of ARN, a better understanding of the disease is required. Animal studies provide crucial insights into disease pathology and the development of therapeutic treatments in a complex in vivo system. The von Szily model, established in 1924, has been a cornerstone for investigating ARN pathogenesis [65]. In this model, HSV-1 is injected into the anterior chamber of one eye in BALB/c mice, leading to inflammation in the injected eye and retinitis in the contralateral eye by 7–10 days post-infection due to viral spread and a delayed hypersensitivity response [66]. This model shows a broad upregulation of inflammatory genes, notably TNF-α, within the posterior segment, like that found in humans [67,68]. Viral spread occurs from the injected eye to the brain and contralateral eye via the optic nerve without the development of clinical encephalitis [69,70,71]. However, more virulent HSV-1 strains result in bilateral retinitis, encephalitis, and death due to faster and more widespread viral replication in the brain, overwhelming innate immune defenses [66,72,73]. In another model and one developed within our lab, HSV-1 is injected into the subretinal space of rabbits or C57Bl/6J mice, resulting in retinal whitening and full-thickness necrosis reminiscent of human disease [74,75]. A recent study found that type I interferon receptor-deficient (IFNAR^−/−^) mice exhibited a significantly worse retinal pathology and higher viral titers than wild-type mice. Additionally, these mice had higher viral titers in the brain and a greater frequency of encephalitis, underscoring the model’s relevance for studying clinical encephalitis in ARN. This suggests that type I interferons are critical in containing HSV-1 within the retina and preventing its spread to the brain [75].

For CMV retinitis, many research groups have attempted to develop translatable models that reflect this disease. Initially, developing an animal model for HCMV retinitis in immunocompetent mice was largely unsuccessful, as only murine CMV (MCMV) antigens or DNA were detected in the ocular tissues of the anterior chamber without retinal involvement [76,77]. Later, the Holland lab created a model using Swiss Webster mice immunosuppressed with cyclophosphamide and injected intravitreally with MCMV, which led to focal retinal necrosis in a few mice [78]. However, many mice died from systemic MCMV infection. A more reproducible model was later developed by injecting MCMV into the supraciliary space of immunosuppressed BALB/c mice, resulting in full-thickness necrotizing retinitis in 80–100% of animals by 10 days post-infection [79,80,81,82]. This model mirrored AIDS-related HCMV retinitis histopathology, including retinal necrosis, cytomegalic cells, and viral inclusions, but failed to replicate the immune profile of HIV-induced AIDS [13,79,82,83]. Specifically, the model lacked the characteristic Th1-to-Th2 cytokine shift and immune cell involvement seen in AIDS [84,85]. To overcome this, Dix and colleagues created a model with the use of C57BL/6 mice infected with the murine retrovirus mixture LP-BM5, which induced murine AIDS (MAIDS), and injected MCMV into the subretinal space of the MAIDS mice, leading to reproducible retinitis similar to that seen in human HCMV retinitis. Unlike corticosteroid-immunosuppressed mice, the MAIDS mice exhibited significant macrophage and neutrophil infiltration in the retina, closely resembling the immune response in AIDS-related HCMV retinitis [86,87].

Though efforts have been made to find translatable in vivo models that represent the clinical pathophysiology of ARN or CMV retinitis, there are still many unknowns regarding the precise mechanisms of the viral pathogenesis and host–virus interactions within the retina. The viral latency and reactivation triggers remain unclear, especially in immune-privileged areas like the retina. Additionally, the role of inflammation in exacerbating retinal damage and the balance between viral replication and immune responses are not fully understood. Early biomarkers for detection and differentiation between causative agents are lacking. Furthermore, the genetic factors that may predispose individuals to severe disease, particularly in immunocompetent populations, are poorly studied. Limitations in current animal models hinder deeper investigation into these issues, and long-term outcomes such as retinal scarring and the potential for regeneration remain largely unexplored.

## 4. Herpes Viruses and Their Roles in Retinitis Progression

The *Herpesviridae* family consists of enveloped, double-stranded DNA viruses with a T = 16 icosahedral capsid symmetry, primarily infecting animal hosts, including humans. Over 90% of adults have been exposed to at least one of the nine known human herpesviruses (HHVs), ranging from HHV-1 to HHV-8 [88,89,90,91]. These viruses can establish a lifelong latency in hosts that may cause recurrent lytic infections that destroy infected cells. The common human herpesviruses include the herpes simplex viruses (HSV)-1 and -2, which usually cause mucocutaneous lesions in the oral or genital areas and occasionally lead to meningoencephalitis. Other viruses like the varicella-zoster virus (VZV), human cytomegalovirus (HCMV), Epstein–Barr virus (EBV), and Kaposi’s sarcoma herpesvirus (KSHV) also contribute to a range of diseases. For instance, HCMV infections are typically asymptomatic in children but can cause severe complications in pregnant women, transplant recipients, and immunocompromised individuals. Herpesviruses, particularly HSV-1 and -2, VZV, and CMV, are implicated in various ocular diseases, including retinitis [92].

### 4.1. Herpetic Simplex Virus (HSV) and Varicella Zoster Virus (VZV)

HSV and VZV are neurotrophic alpha herpesviruses capable of inducing central nervous system (CNS) diseases due to their neuroinvasive and neurovirulent properties. These viruses are highly prevalent. HSV-1 has affected about 67% of the global population, estimated at 3.7 billion people in 2016, whereas HSV-2 has affected around 13%, or approximately 500 million people [93]. HSV-1 is commonly contracted in early childhood via the orolabial route, while HSV-2 is typically acquired through sexual contact later in life [88,91,93]. Initially, HSV infects the epithelial cells of the skin or mucosa, then migrates to establish latency in peripheral nervous system neurons [94,95,96,97]. For VZV, before vaccines were developed, there were about 4 million estimated infected cases in the U.S. in the 1990s, and the mortality rate was high in children [98]. VZV infection often manifests as chickenpox in early childhood. However, developing vaccinations and vaccination campaigns have drastically decreased this incidence, with about 150,000 cases detected each year, according to the CDC [99,100,101]. In immunocompetent individuals, the presence of HSV/VZV in the eye triggers a pronounced immunologic response when it does occur. This reaction involves the infiltration of the vitreous humor and retina by mononuclear cells, predominantly composed of CD4+ T cells, which comprise 70% of the lymphocytes isolated from vitreous samples of acute retinal necrosis cases [102]. Such immune activation often leads to retinal arteriolar vasculitis, resulting in vascular occlusion and the subsequent necrosis of retinal tissues downstream.

Infection with one HSV type normally induces immunity to prevent re-infections with the same serotype, but not the others [103]. In some cases, HSV-1, -2, and VZV can invade the CNS, causing severe and potentially fatal encephalitis, and, in those with specific innate immune deficiencies, recurrent encephalitis [104,105,106,107,108]. HSV has a large linear double-stranded DNA genome covered in an icosahedral capsid, a tegument protein layer, and an envelope with viral glycoproteins. Virus attachment is mediated by glycoprotein B (gB) and C (gC) binding to glycosaminoglycans, with further binding facilitated by gD interacting with multiple cellular receptors, including HVEM and nectin-1 [109,110]. This specificity in receptor interaction influences viral tropism, with certain receptors like HVEM and nectin-1 being crucial in the cornea and nervous system, respectively. Following receptor binding, gD activation triggers the gH/gL complex to initiate membrane fusion at the plasma membrane or within endocytic vesicles, leading to viral entry [109,110,111]. Additionally, HSV can remain dormant in the corneal nerves, another immunologically privileged site, and may reactivate in ocular cells through mechanisms such as corneal transplants, local reactivation, or anterograde travel along the ophthalmic division of the trigeminal nerve [111,112]. Interactions between the nectin-1 receptor and the viral glycoprotein gD facilitate viral entry via endocytosis into various ocular cell types, including RPE cells [113]. VZV reactivates in about one-third of those previously infected to cause herpes zoster (shingles) and can lead to complications like postherpetic neuralgia, uveitis, and vasculitic stroke [100,114]. The host’s innate and adaptive immune responses are crucial in mitigating disease severity and preventing VZV reactivation. This contrasts with its generally self-limited initial infection in childhood, highlighting the complex interplay between host immunity, viral latency, and reactivation.

As mentioned briefly, these viruses can cause latent infections by incorporating their genetic material into host tissues, predominantly within nerve ganglia like the dorsal root ganglia (DRG) or trigeminal ganglia (TG) [97,115]. How these viruses establish latency in neuron cells is poorly understood. Studies have suggested that infections at neuronal cell bodies produce infectious viral particles, whereas axonal infections are typically nonproductive unless complemented by a helper virus containing the viral tegument protein (VP) 16 [103,116,117,118]. VP16 plays a crucial role in initiating lytic transcripts and preventing the silencing of viral gene expression. Interestingly, VP16 travels independently to the nucleus, but may not reach the neuronal nucleus due to the distance from the neurite end. This leads to potential latency due to insufficient levels of VP16 and other proteins essential for active viral replication [103,116,117,118]. Further research is necessary to understand the exact role of VP16 in transitioning from latency to active viral replication, highlighting its importance in viral dynamics and reactivation. HSV- and VZV-related retinal infections, such as ARN, most likely occur due to viral reactivation from sensory ganglia rather than primary infection in adults. While HSV-1, HSV-2, or VZV are typically implicated, neonatal HSV-2 infections have also been identified as potential etiological factors [119,120]. From a retinal perspective, the debate continues over whether ARN development results from viral reactivation or a primary infection event with dissemination and spread from circulating blood.

### 4.2. Cytomegalovirus (CMV)

Cytomegalovirus (CMV), a member of the beta subtype of the *Herpesviridae* family, exhibits morphological similarities to other herpesviruses such as HSV and VZV and shares comparable pathogenic mechanisms. However, CMV more frequently impacts immunocompromised individuals. During a CMV infection, the virus penetrates the eye hematogenously via a compromised BRB [121]. It initially targets retinal microvascular endothelial cells before spreading to adjacent perivascular glia, Müller cells, and RPE cells. The virus infects through the apical membrane of the RPE and spreads laterally from cell to cell. This viral invasion prompts infected endothelial cells to undergo apoptosis, releasing pro-inflammatory mediators from neuro-sensory and glial cells and sensitizing retinal cells to undergo FasL-mediated apoptosis [122,123].

As previously mentioned in Section 3, CMV retinitis generally progresses slower than ARN. A key question in the field is why the progression rate differs so markedly between types of viral retinitis, especially since ARN, PORN, and CMV retinitis all arise from viruses within the *Herpesviridae* family. Furthermore, it is unclear why CMV retinitis, which typically affects only immunocompromised individuals, progresses slower than ARN, which occurs in immunocompetent patients. The potential role of differences in the innate immune response in influencing the progression and inhibition of these viral infections remains an underexplored area of research.

### 4.3. Innate Immune System Evasion Strategies Utilized by Herpesviruses

**Figure 3:**
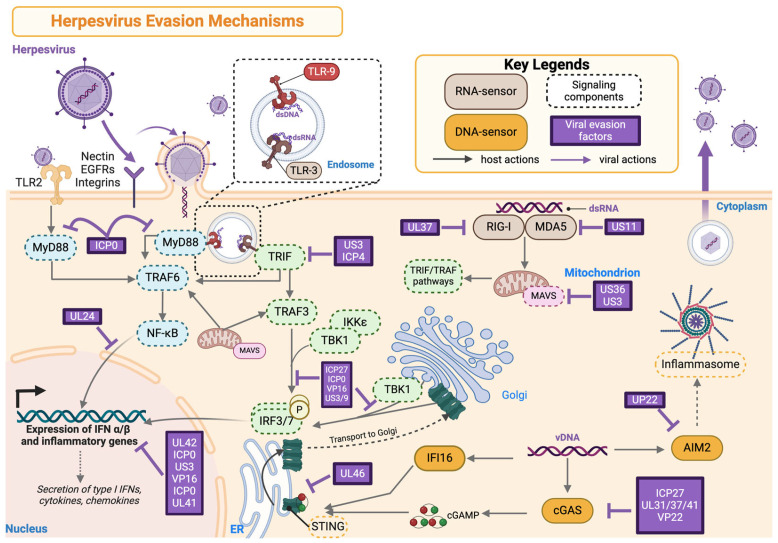
Evasion mechanisms utilized by Herpesviruses. Herpesviruses have evolved specific mechanisms to inhibit multiple stages/responses of host innate immunity to reduce antiviral and proinflammatory chemokine and cytokine production to promote virus survival. Lines with arrows represent host pathways. Lines with blunt end represent protein targets of herpesviruses. This figure was created using Biorender.com (accessed 1 December 2024).

Like many viruses, herpesviruses employ various strategies to evade the host’s innate defenses. These include avoiding detection by pattern recognition receptors (PRRs), blocking their action, inhibiting signaling pathways, and suppressing antiviral gene expression and programmed cell death (PCD). The mechanisms that herpesviruses use to undermine the host immune system have been extensively reviewed [94,124,125,126,127]. This review will highlight a few strategies that herpesviruses use to evade innate immune responses that could potentially impact nervous system immunity. These insights may also be relevant for the retina, where significantly less is known about innate immune responses. A summary of these strategies is provided in Figure 3.

As mentioned in Section 2, PRRs, such as TLRs, are critical in recognizing virus PAMPs. Upon herpesvirus infection, these membrane-spanning proteins recruit adaptor molecules like MyD88, TRIF, and TRAP, initiating signaling cascades that activate transcription factors such as NF-κB and interferon regulatory factor (IRF)3/7. Together, these signaling events stimulate the production of pro-inflammatory cytokines and chemokines, including type I interferons (IFNs), which are key to antiviral defenses [128,129,130]. In the CNS, TLR2 and TLR4 are on the cellular surfaces, while TLR3, TLR7, and TLR9 are within the endosomes [129,131,132]. These receptors detect specific viral components, such as herpesvirus glycoproteins (e.g., gB, gD, and gC) and virion tegument proteins (e.g., US2 and US3), allowing the immune system to recognize and respond to infection [131,132,133,134]. Importantly, the unique immune-privileged status of the eye, combined with its specialized tissue architecture, may lead to different immune responses to herpesviruses compared to other neural tissues [107,135].

While direct evidence in retinal cells is limited, herpesviruses are known to subvert PRR-mediated pathways in other cell types to evade host detection. For example, the alphaherpesvirus protein US3 plays a key role in blocking type I and II interferon (IFN) responses. Seminal studies by Piroozmand et al. and Peri et al. showed that HSV-1 US3 could manipulate innate immune responses, and the absence of this viral protein resulted in an increased sensitivity to IFN-α [136] and the stronger activation of IRF-3 and type I IFN mRNA expression [137]. Additional studies have shown that HSV-1 US3 inhibits IFN-β production [138] and blocks IFN-γ-induced ISG expression [139] by hyperphosphorylating IRF3 or by inhibiting the posttranslational modification of the IFN-γ receptor’s α subunit, respectively. Moreover, Wang et al. found that US3 significantly downregulates NF-κB activation by inhibiting the tumor necrosis factor (TNF)-α-induced nuclear translocation of p65, thereby reducing inflammatory chemokine expression [140]. Another HSV-1 protein, the immediate-early protein (ICP0), can suppress TLR2-mediated innate immune responses and NF-κB signaling [141]. Similarly, HCMV, part of the *Betaherpesviridae* subfamily, uses proteins like US7 and US8 to destabilize TLR3 and TLR4, promoting viral evasion through interactions with host ER-related degradation components such as Derlin-1 and Sec61 (Figure 3) [142]. Additionally, herpesviruses exploit noncoding RNA such as microRNA (miRNA) and long-noncoding RNA (lncRNA) to interfere with host gene regulation. HSV-1 encodes miRNA-373, which targets IRF1, suppressing ISG expression and promoting viral replication [143], while HCMV encodes miR-UL112-3p, which downregulates TLR2 expression, hindering signal transduction in the TLR/NF-κB axis and reducing the expression of pro-inflammatory cytokines such as IL-1β, IL-6, and IL-8 [144]. Notably, recent studies by Shirahama and colleagues on lncRNA *U90926* showed upregulation in a murine retinal photoreceptor cell line post-HSV-1 infection, and the downregulation of this viral RNA resulted in less HSV-1 replication and proliferation [145], while human *U90926* orthologs are highly elevated in the vitreous fluid in patients with acute retinal necrosis and higher levels of expression are associated with a worse visual prognosis [146].

Another strategy that herpesviruses utilize to evade nervous system immunity is inhibiting glial cell DNA sensors. Glial cells such as microglia utilize intracellular DNA sensors such as cGAS and STING to detect viral DNA and trigger antiviral responses upon viral infection [147,148,149,150]. HSV-1 can block the cGAS–STING pathway by expressing proteins like ICP0 and ICP27, which can interfere with the cGAS–DNA binding process, preventing the activation of the downstream signaling that would normally lead to the production of IFNs and other antiviral molecules (Figure 3) [46,151,152]. Similarly, human CMV (HCMV) can block cGAS–STING by synthesizing proteins (e.g., UL31, UL35, UL131, UL122 (IE2), and Us9), which then interact with components of the DNA-sensing machinery, preventing the production of downstream antiviral molecules like IFN [150,153,154,155,156]. Another way is that HSV-1 synthesizes the UL41 gene, which encodes for the viral host shutoff protein (vhs) that degrades host mRNA, including those that encode components of DNA sensing pathways, to reduce the immune response in the CNS [157]. The shutoff of host protein synthesis is unavailable in beta herpesviruses, including HCMV.

Programmed cell death (PCD), such as apoptosis, necroptosis, and proptosis, is often considered to be an autonomous innate cellular response to invading pathogens that can clear the infected cells, reduce viral replication reservoirs, and limit subsequent pathogen invasion. This is best epitomized in patients and mice that develop herpetic encephalitis due to inborn errors of receptor-interacting protein kinase (RIPK) 3, a ubiquitous cytoplasmic cell death regulator that results in impaired neuronal cell death and enhanced HSV-1 growth [158,159]. Hence, another tactic that herpesviruses can utilize to undermine the host defense is expressing factors that dampen the PCD signaling pathways [160,161,162,163]. HSV-1 expresses viral proteins such as ICP4 and ICP27, which inhibit pro-apoptotic proteins like caspases and Bax/Bak, preventing apoptosis in infected cells [164]. Similarly, VZV expresses ORF63, which blocks apoptosis in neurons, aiding in viral persistence within the CNS [165]. HSV-1 also inhibits necroptosis through the ICP6 protein, a viral homolog of the RIP homotypic interaction motif (RHIM), which disrupts the formation of the RIP1–RIP3 complex, a critical step in necroptotic signaling [166]. This inhibition allows viral replication to continue while preventing cell lysis and inflammation.

CMV also encodes various gene products to suppress PCD activity [167]. Proteins like UL37 and UL38 inhibit mitochondrial depolarization and caspase activation, while the viral mitochondrial inhibitor of apoptosis prevents cytochrome c release, blocking the intrinsic apoptotic pathway (Figure 3) [168,169]. To avoid necroptosis, CMV expresses UL36, which blocks RIPK1 activation [170]. Additionally, a recent study by Deng et al. showed that the mouse CMV M84 protein inhibits pyroptosis and the release of proinflammatory cytokines like IL-1β and IL-18 to promote viral replication [171].

## 5. Conclusions

Epidemiological and experimental studies highlight significant knowledge gaps in understanding how the innate immune system responds to herpesvirus infection. It is still unclear whether viral evasion strategies operate in the retina, similar to those in other systems like the brain. The current data point to several key challenges for future research, as follows: (1) identifying in vivo models that reflect clinical presentation, (2) investigating the viral and host-related factors that determine the frequency and severity of viral spread to the retina, and how these contribute to retinitis conditions such as ARN, PORN, or CMV retinitis, and (3) developing and testing new strategies to prevent viral reactivation and spread in the retina, in order to minimize retinal damage and promote improved visual outcomes. While most of this future work will require in vivo or ex vivo systems due to the complex host–virus immunological-based interactions in a multifaceted tissue not easily replicated, the creation of in silico or in vitro models may help to identify drug targets and expedite drug development, which is notoriously slow and labor-intensive in animal models. These new agents, we hope, will reduce the development of immunopathology by targeting specific inflammatory proteins and pathways, inhibit viral replication, and ultimately, improve visual outcomes in ARN. In the interim, a clinical approach that utilizes intravitreal and systemic antivirals with topical and systemic steroids will remain the current therapeutic interventions for this devastating and potentially deadly disease.
